# One Health approach to identify research needs in bovine and human babesioses: workshop report

**DOI:** 10.1186/1756-3305-3-36

**Published:** 2010-04-08

**Authors:** Adalberto A Pérez de León, Daniel A Strickman, Donald P Knowles, Durland Fish, Eileen Thacker, José de la Fuente, Peter J Krause, Stephen K Wikel, Ryan S Miller, Gale G Wagner, Consuelo Almazán, Robert Hillman, Matthew T Messenger, Paul O Ugstad, Roberta A Duhaime, Pete D Teel, Alfonso Ortega-Santos, David G Hewitt, Edwin J Bowers, Stephen J Bent, Matt H Cochran, Terry F McElwain, Glen A Scoles, Carlos E Suarez, Ronald Davey, Jeanne M Howell Freeman, Kimberly Lohmeyer, Andrew Y Li, Felix D Guerrero, Diane M Kammlah, Pamela Phillips, Joe M Pound

**Affiliations:** 1 Knipling-Bushland U.S. Livestock Insects Research Laboratory, USDA-ARS, Kerrville, TX, USA; 2 National Program, Animal Production and Protection, USDA-ARS, Beltsville, MD, USA; 3 Animal Disease Research Unit, U.S. Department of Agriculture/Agriculture Research Service, Pullman, WA 99164-7030, USA; 4 Department of Epidemiology and Public Health, Yale School of Medicine, New Haven, CT 06520, USA; 5 Department of Veterinary Pathobiology, Center for Veterinary Health Sciences, Oklahoma State University, Stillwater, OK 74078, USA; 6 Instituto de Investigación en Recursos Cinegéticos IREC (CSIC-UCLM-JCCM), Ronda de Toledo s/n, 13005 Ciudad Real, Spain; 7 Division of Epidemiology of Microbial Diseases, Yale School of Public Health, New Haven, CT 06520-8034, USA; 8 Department of Pathology, University of Texas Medical Branch, Galveston, TX 77555, USA; 9 USDA-APHIS, 2150 Centre Ave, Bldg. B-2W4, Fort Collins, Colorado 80526, USA; 10 Department of Veterinary Pathobiology, College of Veterinary Medicine, University Drive and Agronomy Road, Texas A&M University, College Station, TX 77843, USA; 11 Facultad de Medicina Veterinaria y Zootecnia, Universidad Autónoma de Tamaulipas, Km. 5 carretera Victoria-Mante, CP 87000 Ciudad Victoria, Tamaulipas, Mexico; 12 Texas Animal Health Commission, Austin, TX 78758-4013, USA; 13 Cattle Fever Tick Eradication Program, USDA-APHIS-VS, Riverdale, MD 20737, USA; 14 USDA-APHIS-VS, Thornberry Bldg., Rm. 220 903 San Jacinto Blvd. Austin, TX 78701, USA; 15 Cattle Fever Tick Eradication Program, USDA-APHIS-VS, San Juan, TX 78589, USA; 16 Department of Entomology, Texas A&M University, College Station 77843, USA; 17 Animal & Wildlife Sciences, Texas A&M University, 700 University Blvd., Kingsville, Texas 78363, USA; 18 Cattle Fever Tick Eradication Program, USDA-APHIS-VS, Laredo, TX 78040, USA; 19 Animal Disease Research Unit, Agricultural Research Service, United State Department of Agriculture, Pullman, WA 99164-6630, USA; 20 Program in Vector-Borne Diseases, Department of Veterinary Microbiology and Pathology, Washington State University, Pullman, WA 99164-7040, USA; 21 Animal Diseases Research Unit, Agricultural Research Service, U.S. Department of Agriculture, Pullman, Washington 99164, USA

## Abstract

**Background:**

*Babesia *are emerging health threats to humans and animals in the United States. A collaborative effort of multiple disciplines to attain optimal health for people, animals and our environment, otherwise known as the One Health concept, was taken during a research workshop held in April 2009 to identify gaps in scientific knowledge regarding babesioses. The impetus for this analysis was the increased risk for outbreaks of bovine babesiosis, also known as Texas cattle fever, associated with the re-infestation of the U.S. by cattle fever ticks.

**Results:**

The involvement of wildlife in the ecology of cattle fever ticks jeopardizes the ability of state and federal agencies to keep the national herd free of Texas cattle fever. Similarly, there has been a progressive increase in the number of cases of human babesiosis over the past 25 years due to an increase in the white-tailed deer population. Human babesiosis due to cattle-associated *Babesia divergens *and *Babesia divergens*-like organisms have begun to appear in residents of the United States. Research needs for human and bovine babesioses were identified and are presented herein.

**Conclusions:**

The translation of this research is expected to provide veterinary and public health systems with the tools to mitigate the impact of bovine and human babesioses. However, economic, political, and social commitments are urgently required, including increased national funding for animal and human *Babesia *research, to prevent the re-establishment of cattle fever ticks and the increasing problem of human babesiosis in the United States.

## Background

Babesioses are emerging tick-borne diseases in humans and animals caused by the intraerythrocytic apicomplexan protozoa *Babesia *spp [1]. More than 100 species of *Babesia *have been described, several remain to be fully described, and it is likely that many more species remain to be discovered [2]. While ticks are second only to mosquitoes as worldwide vectors of human diseases, they are the most relevant vectors of disease-causing pathogens in domestic and wild animals [3]. Climate, host movement, animal husbandry practices, vector distribution and vector population changes affect the epidemiology of babesioses and other tick-borne diseases. Changes in these factors could result in enhanced *Babesia *transmission across vertebrate species by infected ticks and a greater role of certain wildlife in amplifying tick vector populations [4].

The One Health concept, which is used here to define the collaborative effort of multiple disciplines to attain optimal health for people, animals and our environment, was applied to a workshop organized to identify gaps in the scientific knowledge regarding bovine and human babesioses in the United States [5]. Emphasis was placed on the potential threat of reintroduction of cattle fever ticks (CFT) into the U.S. and concomitant increase in the risk for outbreaks of bovine babesiosis, but we also addressed the emerging problem of human babesiosis because collaboration between entomologist, epidemiologists, physicians, veterinarians, and related health-sciences experts can most effectively address these closely related health issues.

Discussions by the workshop participants focused on (I) epidemiology and surveillance, (II) ecology and biology of tick vectors and wildlife, (III) diagnosis, treatment, and prevention, (IV) integrated approaches for sustainable CFT eradication, and (V) tick vaccines in the context of bovine and human babesioses. Here, we present a list of research needs for bovine and human babesioses as the outcome of the workshop exercise. The translation of this research is expected to provide veterinary and public health systems with the tools to mitigate the impact of tick-borne babesioses.

## Bovine babesiosis

Bovine, canine, and equine babesioses are among the most economically relevant infections of domestic animals. Infestations with CFT, *Rhipicephalus *(*Boophilus*)*microplus *and *R*. (*B*.)*annulatus*, economically impact cattle production in tropical and subtropical regions of the world. They cause damage directly by reducing weight gain and milk production and are vectors of pathogens that cause bovine babesiosis (*Babesia bovis *and *B. bigemina*), also known as cattle tick fever or Texas cattle fever, and the etiologic agent of anaplasmosis (*Anaplasma marginale*) [4,6,7]. The U.S. Cattle Fever Tick Eradication Program (CFTEP) was initiated in 1906 to free the national cattle herd from bovine babesiosis [8]. Subsequently, CFT were officially eradicated from the U.S. in 1943 with the exception of a permanent quarantine zone that remains in place today in South Texas along the border with Mexico. It is estimated that the livestock industry realizes annual savings of at least 3 billion dollars at today's currency rate since the U.S. was declared free of CFT and bovine babesiosis [8]. However, the apparent re-infestation of the U.S. by CFT, which is thought to be due primarily to changes affecting the ecology of ticks and wild ungulate hosts that may support the maintenance and dissemination of CFT, is impacting the ability of state and federal agencies to keep the national herd free of CFT and consequently bovine babesiosis.

### Epidemiology and surveillance

**Figure 1:**
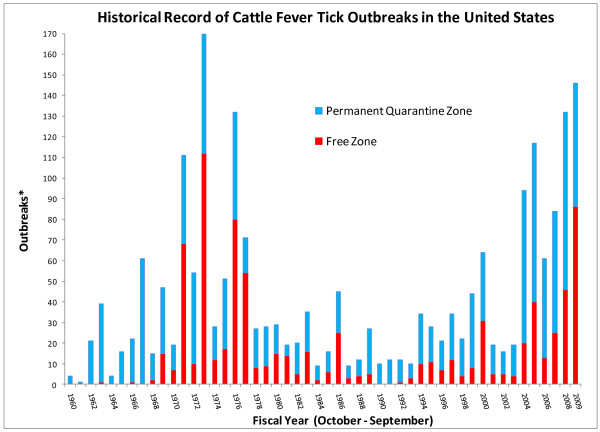
**Historical record of cattle fever tick outbreaks in the United States**. *Each fiscal year shows the contribution by the Permanent Quarantine and Free zones to the total number of outbreaks. The Free Zone comprises the area in the forty-eight contiguous states outside the Permanent Quarantine Zone, which is located along the Rio Grande River in southern Texas. The period shown in the graph depicts records maintained by APHIS-VS and it covers fiscal years 1960 through 2009. Data updated after [59].

The number of CFT outbreaks within and outside the permanent quarantine zone fluctuates with time (Fig. 1). A significant incursion of CFT took place in the 1970s. From a total of 170 outbreaks recorded in 1973, 112 occurred outside of the permanent quarantine zone. It took six years to re-eradicate the ticks at a significant cost to producers and agencies with mandated control responsibilities. During the last five years, the level of CFT activity in the U.S. has once again increased substantially. The largest number of infested premises in the permanent quarantine zone was initially recorded in 2005, but that record was broken again in 2008 when CFT were detected in 85 premises. A sustained spillover of CFT into the free zone also has been noted since 2004. There appears to be a spike in the number of outbreaks in the permanent quarantine zone prior to the explosion of outbreaks in the free zone that was recorded in 1973 (Fig. 1). Not only do we see a similar pattern now, but also the development of outbreaks in the permanent quarantine zone suggests that the number and extent of outbreaks in the free zone may continue to increase (Fig. 1). This situation is indeed an emergency that requires both immediate and long-term interventions.

Surveillance is critical to determine if changes in environmental factors are affecting tick populations and/or pathogen transmission dynamics [9]. Regulations for CFT surveillance are established and applied to domestic cattle populations. Current surveillance for CFT is designed for detection of infestations on cattle moved into the U.S. But, the landscape in South Texas has changed considerably in the recent past. For example, the number of non-traditional farms and properties maintained exclusively for hunting has increased substantially. These factors, combined with the increased densities of native (i.e. white-tailed deer), and non-native (exotic) ungulate species, have complicated surveillance. White-tailed deer and exotic ungulate species may support CFT populations in the absence of cattle, and this may add a new dimension to tick population dynamics in this region. However, adequate surveillance tools and methodologies have not been developed for native and exotic ungulate species. The lack of sufficient surveillance tools for detection of CFT on alternative hosts in the absence of cattle is urgently needed to maintain a successful eradication program.

### Ecology and biology of tick vectors and wildlife

Native and non-native species of wild ungulates, particularly white-tailed deer, are a major complicating factor in CFT eradication efforts. CFT eradication in pastures vacated of cattle was considered impossible as long as white-tailed deer remained within an area [10]. Approximately 20,000 white-tailed deer had to be culled (Hourrigan, unpublished data) before CFT eradication in Florida was officially completed in 1961 [11]. The suitability of cervid and non-domestic bovid species as hosts for CFT is well documented [12-15]. In South Texas, complexities of the expanding infestations are compounded by agricultural practices, as well as recreational, environmental and ecological conditions promoting an abundance of white-tailed deer and non-native wild ungulates. Hunter-killed wildlife surveillance in Zapata and Starr Counties revealed that during fiscal year 2009 at least 30% of all the CFT infestations in the permanent quarantine zone occurred in white-tailed deer. Additionally, Cantu et al. [16,17] documented the exposure of white-tailed deer to *B.bovis *and *B.bigemina*. These findings highlight the need to understand risks to cattle populations from CFT infestations and *Babesia *infection in native and non-native ungulate populations.

To date, only one non-native ungulate species, the nilgai antelope (*Boselaphus tragocamelus*), has been evaluated for host suitability [14]. Non-native ungulate populations have expanded considerably in Texas and other states over the last 30 years. Texas is estimated to have more than 70 species of non-native ungulates, numbering over 200,000 animals [18]. Incomplete fencing and poor maintenance of high fences has allowed many animals to escape, and at least five species of exotic ungulates exist as free ranging populations in Texas and other regions of the U.S. where they are commonly farmed or have been intentionally released [18-26]. These animals pose a continued risk for maintaining and disseminating CFT populations.

Some species of non-native wild ungulates may also contribute to the re-establishment of CFT infestations and Texas cattle fever. Two separate efforts conducted in 2009 confirmed that a herd of nilgai antelope in Cameron County, Texas was infested with CFT. Additionally, nilgai were reported to be susceptible to infection with *Babesia *spp. in India [27].

The roles that white-tailed deer and non-native ungulate species play in the dissemination of CFT across the landscape are poorly understood. In addition, it is unclear how this potential dissemination of CFT may be linked to current and past infestations of cattle.

A small number of studies have been published on cattle-CFT interactions during the blood-feeding process [28,29]. Results from these studies will advance our understanding of the immunological basis for innate and acquired resistance to CFT in cattle. However, the molecular basis and effects on host defense mechanisms of bioactive factors in the saliva of CFT remains largely unexplored. Much work needs to be done to provide the foundation for development of novel control strategies for disease prevention.

### Diagnosis, treatment, and prevention

Various assays have been developed and used for diagnostic purposes in research on Texas cattle fever [30,31]. Complement fixation, indirect fluorescent antibody and polymerase chain reaction tests are adequate for early detection and can be used for long-term carriers. Competitive ELISA yields positive results, but this technological platform is not currently commercially available [30,31]. Cattle imported from Mexico are not tested for *Babesia *because the tick vector had been contained within the permanent quarantine zone and current CFT eradication measures appear to have prevented outbreaks of clinical Texas cattle fever. However, the emerging re-infestation by CFT increases the risk for the re-emergence of Texas cattle fever in the U.S. CFT eradication achieved since 1943 also obviated the need to use babesiacidal drugs in cattle. Thus, there is no drug registered in the U.S. to treat bovine babesiosis. The risk is amplified further by the suspected ability of wildlife to serve as competent reservoirs for *B.bovis *and *B. bigemina *[17,27].

Tick "scratching" is the method approved to detect and identify CFT infestations in cattle and deer. However, larvae infesting animals can readily escape human detection. Serologic tests and other sensing technologies should be explored for their utility to test cattle and wildlife for CFT infestation. For example, antibodies to unique salivary proteins in other tick vectors can be detected by ELISA [32]. This technique may allow larval detection and an ELISA test would be amenable to standardization. However, tick "scratching" yields immediate results on site, while an ELISA would require dedicated equipment and results take longer to develop. In addition, serologic assays might not be able to distinguish between current and previous infestations. Increased efficiency for CFT detection may be achieved through the combination of different methods. "Scratching" of live white-tailed and other wildlife is more problematic. This is especially challenging in areas devoid of cattle that are adjacent to quarantined premises. The challenge is complicated further by the lack of a sentinel system for the presence of CFT in the landscape when the option to vacate cattle from premises is exercised by the landowner.

*Babesia *vaccines against *B. bovis *and *B. bigemina *consisting of living attenuated parasites are in use in endemic areas worldwide. However, live *Babesia *vaccines have a number of limitations, including short shelf life, possibility of reversion to virulence, the need of a cold chain, and the establishment of persisting *Babesia *infections in the vaccinated areas. Additionally, vaccination with live vaccines causes the animals to become seropositive against *Babesia *thus making difficult to distinguish vaccinated from infected animals upon serological screening, confounding diagnostic efforts. Ideally vaccines should overcome these limitations, and preferably be based on strains that are not transmissible by ticks. In the context of the current increased risk of a *Babesia *outbreak that might cause substantial economic damage, it would be practical to have both *B. bovis *and *B. bigemina *live vaccines, or at least vaccine-ready attenuated strains, available in case urgent preventive measures are required. However, such vaccines are not licensed for use in the U.S. currently. Research efforts are currently being directed towards the use of a novel transfection system to aid in the development of better defined non tick-transmissible vaccine strains that may overcome some of the limitations of current vaccines [33]. Together with the availability of the *B. bovis *genome [34], *Babesia *transfection techniques can be helpful for: (i) helping to define virulence factors by gene knock-out techniques; (ii) the production of viable and attenuated *Babesia *strains that are deficient in known virulence factors; (iii) introducing antigenic molecular markers that may help to discriminate vaccinated from naturally infected animals; (iv) producing *Babesia *strains that express protective tick antigens, such as Bm86, that may elicit dual protection, against *Babesia *disease and tick infestation. Effective subunit vaccines would be ideal for prevention of bovine babesiosis but no one is yet available [35]. Developing subunit vaccines will likely require better characterization of protective immune responses, the host-parasite relationship, further identification of vaccine candidate antigens, and effective methods of vaccine delivery [35].

### Integrated approaches for sustainable cattle fever tick eradication

Cattle drives in the nineteenth century facilitated the expansion of CFT populations throughout the U.S [36]. The former geographic range of CFT in the U.S. covered 14 southern states including Missouri, Kentucky and southern California. In 1868, a major outbreak of cattle tick fever killed 15,000 cattle in Illinois and Indiana following the importation of apparently healthy cattle from Texas [37]. Following definitive studies done in collaboration with Frederick L. Kilborne which lead to our understanding of how the disease was transmitted to cattle by CFT, Theobald Smith stated, *"Eliminate the ticks on cattle and you eradicate the ticks because they cannot live elsewhere" *[38]. That concept is untenable today despite heroic efforts by Cattle Fever Tick Eradication Program personnel operating with limited resources and is inadequate to sustain CFT eradication in the U.S.

Bovine babesiosis and CFT are endemic in Mexico. Almost a million cattle are imported annually into the U.S. from Mexico through Texas. Stray animals, smuggling of livestock, free crossing of wildlife across the U.S. - Mexico border, and pervasive acaricide resistance in Mexico are major risk factors for re-emergence of CFT and bovine babesiosis in the U.S. The organophosphate compound Coumaphos is the only acaricide approved for official use by the Cattle Fever Tick Eradication Program in dipping vats since 1970. Another challenge for total eradication is that CFT have no natural enemies in the U.S. since they are an invasive species that was brought into the country with livestock by European settlers.

Despite recent technological advancements that fueled the development and commercialization of vaccines based on the tick antigen Bm86 in Australia and certain Latin American countries [39], acaricides remain the principal means commercially accessible for tick eradication. However, development and commercialization of novel acaricides is challenging and costly [40]. This situation reinforces the need for alternative approaches to control tick infestations [41,42].

### Tick vaccines

Tick vaccines have been developed that induce immunological protection of vertebrate hosts against tick infestations. The feasibility of controlling tick infestations through immunization of hosts with selected tick antigens was demonstrated with the development of vaccines based on recombinant Bm86 protein derived from the gut of *Rhipicephalus (Boophilus) *spp. and used to induce a protective immune response that reduced infestations on cattle [39,41,42]. These products represent the first generation of tick vaccines to be commercialized. Control of ticks by vaccination has the advantages of being cost-effective, reducing environmental contamination and prevention of the selection of drug resistant ticks that result from repeated acaricide application. Development of vaccines against ticks would also allow for inclusion of multiple antigens that could target a broad range of tick species and may also prevent pathogen transmission. Vaccines that target tick saliva molecules essential for successful blood feeding and pathogen transmission are an active area of research.

The utility of tick vaccines based on Bm86, used alone or in combination with acaricides, for eradication purposes needs to be explored. Gavac^® ^is one of the Bm86-based tick vaccines, which is registered and commercially available in Mexico, but not in the U.S. Tick vaccines have to be registered in the U.S. before business concerns can sell them to producers for use in cattle. The development of a critical path for registration and the need for production domestically would need to be considered if Bm86-based tick vaccines show potential as a tool for CFT eradication. Assessing this potential requires the conduct of trials in the U.S. before those anti-tick vaccines are licensed for use in South Texas.

See Table 1 for list of research needs in bovine babesiosis.

**Table 1:** Research needs for bovine and human babesioses by scientific and technological topic.

Topic	Bovine babesiosis	Human babesiosis
Epidemiology & surveillance	Integrate refinement of tick population dynamics models, data gathering, as well as the knowledge of land use, numbers and species of *Babesia *tick vectors and domestic and wildlife hosts with economic analyses of their relevance in order to facilitate and enhance decision-making processes for optimal allocation of resources for regulatory and research programs	Assess current status and forecast future epidemiologic trends of babesioses in humans and keystone hosts through the expansion of field data capture methods and through collaborations between federal and state regulatory agencies and academic institutions

	Refine existing methods and develop new tools for active surveillance, such as serological tests and alternatives or refinement to "scratching" (use of inspector's fingers to feel the skin of the animal in its entirety, from head to tail, searching for ticks) of wildlife to evaluate tick exposure, the detection of *Babesia *in ticks and hosts, and the control of cattle movement	Investigate the tick vectors and pathogenesis of babesiosis caused by *B. microti*, which is the primary etiologic agent of human disease

	Explore genetic structure of CFT populations across the range of outbreaks in South Texas to determine tick geographic origin(s) and population dynamics	Continue studies of *Babesia*-host interactions for infection in humans

	Determine whether CFT from outbreaks in South Texas are infected with *B*. *bigemina *and/or *B*. *bovis *to assess risk potential for outbreaks of clinical infection in the U.S.	Increase studies of transfusion transmitted babesiosis, especially regarding prevention

	Continue studies of *Babesia*-host interactions for infection in cattle and wildlife	

Ecology & biology of tick vectors & wildlife	Evaluate the host suitability of non-native wild ungulates common to South Texas for CFT	Investigate white-tailed-targeted tick control and white-tailed deer population management strategies as a means to decrease the risk for tick-borne babesiosis in humans

	Define the ecological role of native and non-native wild ungulates in maintaining CFT populations in the absence of cattle	

	Conduct ecological studies to determine whether native and non-native wild ungulates play a role in the long term maintenance and dissemination of CFT populations, particularly between infested and tick-free areas	

	Characterize molecular and cellular interactions at the host-blood feeding tick interface for bovine and non-bovine hosts	

	Conduct immunological studies to simultaneously characterize and correlate *R*. *microplus *and *R. annulatus *salivary gland gene expression with host gene expression for infestations with uninfected ticks and for infestations with ticks infected with *B.bovis *or *B.bigemina*	

	Characterize tick-host interactions and tick feeding and developmental time for *R*. *microplus *and *R. annulatus *infestation of white-tailed deer under controlled experimental infestations	

	Determine the developmental periods and survivorship of cattle ticks throughout the year, number of tick generations per year under field conditions, and seasonal dynamics in southern Texas and/or Northern Mexico	

	Determine if pathogen-free *R*. *microplus *and *R. annulatus *can become infected by taking a blood meal from *Babesia*-infected white-tailed deer	

Diagnosis, treatment, & prevention	Improve and make commercially available diagnostic assays to rapidly detect Texas cattle fever and CFT	Improve existing diagnostic tools and develop new assays to detect human babesiosis to improve early diagnosis, better predict disease complications, and screen blood donors for silent infection

	Assess level of preparedness by federal and state regulatory agencies and the national animal health laboratory network to handle an outbreak of Texas cattle fever	Initiate interdisciplinary investigations to define current and ecological and environmental factors associated with changes in tick vector and *Babesia *distributions

	Establish a surveillance program to assess prevalence of *B. bovis *and *B. bigemina *in wildlife and cattle in the permanent quarantine zone	

	Conduct field research with cattle as tracer species in similar way as suggested for the use of other species as sentinels of ecological health [52], to generate science-based information for developing decision-support tools for the CFT eradication program	

	Develop new technologies for CFT surveillance and detection in cattle and wildlife	

	Develop risk assessment systems to evaluate tick dispersal in wildlife species adjacent to infested premises vacated of cattle	

	Continue research to develop and test live attenuated and recombinant vaccines for the control of babesiosis	

Integrated approaches for sustainable CFT eradication	Revisit sterile tick techniques. In the late 1980s, sterile tick experiments were performed on St. Croix with *R. annulatus *× *R. microplus *tick hybrids (R. Davey, unpublished results). One hundred and eighty million hybrid larvae were put in tea bags in the field. When sterile larvae were dropped in the field, the tick population decreased over 7 to 8 months, but then began to increase again. The disadvantages of this method are the difficulties for a laboratory to produce enough sterile larvae, the immobility of ticks and acceptance by owners to put more ticks on their animals. Alternative methods to produce sterile ticks using RNA interference have been proposed [53]. However, this strategy has serious limitations relative to large-scale field trials and practical application on broad geographical areas. However, these new technologies coupled with further observations on crosses between *R. annulatus *and *R. microplus *and between some *R. microplus *geographical strains that produce sterile ticks warrant further studies on the potential use of the sterile tick technique for CFT eradication [[54,55] and references thereof].	Not applicable

	Continue and improve research on the mechanisms and dynamics of tick genetic resistance to acaricides. Apply this knowledge on the enhanced use of acaricides and acaride combinations	

	Develop acaricide combinations. The combination of acaricides and tick growth regulators offers the potential to achieve the high levels of efficacy required for eradication. These efforts need to be pursued despite current challenges to seek and secure approval for registration by the regulatory agencies. The use of novel application technologies like electro-charged spray systems that could improve the spray/dip process should be investigated	

	Investigate passive administration of systemic acaricides for cattle. Mineral/protein blocks are commonly used in cattle production systems. The use of a mineral/protein block medicated with a systemic acaricide such as ivermectin could be a useful tool for the eradication of CFT	

	Develop sustained acaricide delivery systems. When applicable per eradication regulations, the practice of dipping cattle in a vat with Coumaphos every 14 days is both a costly and laborious effort. Thus, systems delivering acaricides in a sustained fashion to achieve eradication efficacy levels for at least 2 months are needed. This approach requires fine-tuning of available formulations and sustained-release technologies to address safety and withdrawal period issues to deliver a registerable and marketable product accessible to the producer. Microspheres and other sustained release technologies have the potential to provide those solutions. However, these formulations face the same commercial barriers mentioned above for traditional acaricides. Business models like the public-private partnership for product development to treat neglected diseases requires consideration as a strategy to achieve solutions for the Cattle Fever Eradication Program involving proven technologies that remain undeveloped and collecting dust on the shelf [56]	

	Research for natural products. Natural products like fungi and botanicals although shown to be potential alternatives [57,58], are not yet available and more research is needed before these products can be considered and integrated into tick control programs	

	Enhance exchange of information between regulatory agencies, research institutions, and the public to facilitate the development and implementation of evidence-based regulations for the CFT eradication program addressing the ecology of wildlife/cattle-tick-*Babesia *interactions	

	Increase efforts and collaborations with agricultural extension systems to disseminate current knowledge and research findings among producers and the public in the U.S. to raise awareness of current national biosecurity threat involving CFT and bovine babesiosis. Additionally, similar efforts need to be established in Mexican states bordering the permanent quarantine zone in Texas through collaboration with colleagues in Mexico	

Tick vaccines	Compare the nucleotide sequences of Bm86 orthologs in U.S. strains of *R. microplus *and *R. annulatus *with those of the commercial Bm86 vaccines to determine whether antigen sources need to be derived from geographic strains	Tick-vaccines and delivery methods for white-tailed deer to prevent human babesiosis

	Identify new tick protective antigens and delivery systems for cattle and wildlife	

	Conduct studies to determine whether the treatment of cattle with Gavac® or other Bm86-based vaccines in Mexico and the U.S. permanent quarantine zone prevents CFT outbreaks in Texas	

## Human babesiosis

Human babesiosis was first reported in 1957, more than a half century after the problem of cattle babesiosis was recognized. Since that time, the epidemiology of human babesiosis has changed from a few isolated cases in coastal New England to the recognition of expanding endemic areas in the northeastern and mid-western United States and episodic cases reported in Europe, Asia, Africa and South America [43]. *Babesia microti*, the most common *Babesia *species that causes human babesiosis, is endemic in much of the Northeast and northern Midwest and is also the most frequent transfusion transmitted microbial agent in the United States [44]. Babesiosis is most often a mild to moderate illness that lasts for about a week. It also may have significant associated morbidity and a mortality rate that ranges from 3-5% in previously health people to greater than 20% in immunocompromised hosts [43,45].

### Epidemiology and surveillance

Babesiosis is an emerging infection among humans. There has been a progressive increase in reported cases each year over the past 25 years in northeastern and northern mid-western states, including recently described cattle-associated *Babesia divergens *and *Babesia divergens*-like cases [43]. No national reporting requirement currently exists and the number of actual cases is thought to be greatly underestimated. *Babesia *infection has also been increasingly identified as a cause of disease in people throughout the world. Severity of infection ranges from asymptomatic to fulminant disease resulting in death [43,45]. The majority of healthy adults experience a mild to moderate illness. Immunocompromised individuals are at the highest risk of severe disease, including those with malignancy, HIV infection, absence of a spleen, use of immune-suppressive drug therapy, and people over the age of 50 years. Asymptomatic carriers present a considerable safety risk to the blood supply and babesiosis is the most commonly reported infectious agent transmitted by blood transfusion in the U.S [46].

### Ecology and biology of tick vectors and wildlife

As with CTF in Texas, the risk of human babesiosis in the northern US in influenced by the distribution and abundance of wildlife species. Rodent reservoirs of *B. mictori *are ubiquitous, but enzootic transmission is only known to occur in the presence of *I. scapularis *[47].

The primary cause for the emergence of human babesiosis is due to an increase in white-tailed deer populations in the Northeast and upper Midwest which host the adult stage of *I. scapularis*. Controlling tick infestations in white-tailed deer or elimination of the deer population may sharply reduce the risk of babesiosis and other I. *scapularis*-borne infections such as Lyme disease and human anaplasmosis. Like other tick-borne disease systems where white-tailed deer are a keystone host, deer-targeted tick control in the Northeast resulted in an overall decrease in the human risk for exposure to *Borrelia burgdorferi *and *Anaplasma phagocytophilum *[48]. Additionally, a population of *I. scapularis *ticks was eliminated after deer were removed from Monhegan Island, Maine [49]. The local elimination of white-tailed deer or methods for preventing *I. scapularis *from feeding upon deer, such as acaricides or anti-tick vaccines, seems to provide the most promising option for preventing human babesiosis as well as other *I. scapularis*-borne pathogens [47].

### Diagnosis, treatment, and prevention

Diagnosis of human babesiosis depends on laboratory testing techniques because symptoms are relatively non-specific. Definitive diagnosis of *Babesia *infection is generally made by microscopic identification of the organism on thin blood smear, amplification of *Babesia *DNA by PCR and detection of *Babesia *antibody by ELISA [50]. Antimicrobial therapy consists of atovaquone and azithromycin or clindamycin and quinine. Only two standard antimicrobial combinations currently exist. The combination of atovaquone and azithromycin is effective and well tolerated and is the most commonly used antibiotic treatment of human babesiosis [51]. Rare clinical resistance in a few immunocompromised hosts has been described. The other combination of clyndamicin and quinine is especially useful in treatment of severe *Babesia *cases, but is often poorly tolerated. Some immunocompromised hosts do not clear infection for months or years despite multiple courses of antibiotics that can result in a mortality rate as high as 20% [45]. Exchange transfusion may be life saving in severe cases. The use of multiple prevention strategies is recommended and consists of personal, residential and community approaches [43].

### Tick vaccines

Babesiosis vaccines have not been developed for humans. Tick vaccines may help control of human babesiosis by reducing the risk of pathogen transmission from animals to humans after vaccination of animal reservoir species.

See Table 1 for list of research needs in human babesiosis.

## Conclusions

Keeping CFT eradicated from the U.S. is a current and critical agricultural biosecurity issue of national relevance. Human babesiosis is an emerging disease of public health concern in the U.S., especially for the immunocompromised and people receiving blood transfusions. The One Health approach to the discussion of current issues on emerging babesioses during the workshop helped identify commonalities in research and development initiatives that are critically important to mitigate their impact on human and animal health. We suggest that research be prioritized to these areas where considerable gaps in knowledge and technology were identified: 1) tick vector ecology studies addressing the epidemiology of human and animal babesioses; 2) the molecular basis of host-*Babesia *and host-tick interactions in humans, livestock and wildlife; 3) implications of the wildlife-livestock interface on the apparent resurgence of CFT outbreaks and the reservoir status of white-tailed deer and non-native wild ungulates for *B*. *bovis *and *B*. *bigemina*; 4) integrated approaches for sustainable CFT eradication to include research on safer acaricides with new modes of action and the development of more effective formulations using active ingredients already registered or approved with the regulatory agencies; 5) diagnostic and prophylactic interventions for control of human and animal babesioses; 6) assessing the utility of prophylactic intervention with Bm86-based vaccines, or through the use of other tick protective antigens, in CFT eradication efforts as well as tick-vaccines and delivery methods for white-tailed deer in human babesioisis prevention. Initiatives to pursue the research needs presented here require adequate funding, but the One Health concept offers the opportunity to focus interdisciplinary research efforts that maximize the use of limited resources through collaborations between investigators with expertise in the human and veterinary medical sciences.

## Competing interests

The authors declare that they have no competing interests.

## Authors' contributions

All coauthors in the paper contributed to drafting the manuscript.
